# Iridescent biofilms of *Cellulophaga lytica* are tunable platforms for scalable, ordered materials

**DOI:** 10.1038/s41598-023-38797-0

**Published:** 2023-08-14

**Authors:** Claretta J. Sullivan, Kennedy Brown, Chia-Suei Hung, Joseph Kuo-Hsiang Tang, Mark DeSimone, Vincent Chen, Pamela F. Lloyd, Maneesh Gupta, Abby Juhl, Wendy Crookes-Goodson, Milana Vasudev, Patrick B. Dennis, Nancy Kelley-Loughnane

**Affiliations:** 1 Materials and Manufacturing Directorate, Air Force Research Laboratory, Wright Patterson Air Force Base, OH 45433 USA; 2 Department of Bioengineering, University of Massachusetts Dartmouth, Dartmouth, MA 02747 USA

**Keywords:** Imaging, Biomaterials - cells, Biomaterials, Bacteriology, Biofilms, Biomaterials, Biomaterials - cells, Soft materials, Liquid crystals, Self-assembly, Biological techniques, Biotechnology, Microbiology, Materials science

## Abstract

Nature offers many examples of materials which exhibit exceptional properties due to hierarchical assembly of their constituents. In well-studied multi-cellular systems, such as the morpho butterfly, a visible indication of having ordered submicron features is given by the display of structural color. Detailed investigations of nature’s designs have yielded mechanistic insights and led to the development of biomimetic materials at laboratory scales. However, the manufacturing of hierarchical assemblies at industrial scales remains difficult. Biomanufacturing aims to leverage the autonomy of biological systems to produce materials at lower cost and with fewer carbon emissions. Earlier reports documented that some bacteria, particularly those with gliding motility, self-assemble into biofilms with polycrystalline structures and exhibit glittery, iridescent colors. The current study demonstrates the potential of using one of these bacteria, *Cellulophaga lytica*, as a platform for the large scale biomanufacturing of ordered materials. Specific approaches for controlling *C. lytica* biofilm optical, spatial and temporal properties are reported. Complementary microscopy-based studies reveal that biofilm color variations are attributed to changes in morphology induced by cellular responses to the local environment. Incorporation of *C. lytica* biofilms into materials is also demonstrated, thereby facilitating their handling and downstream processing, as would be needed during manufacturing processes. Finally, the utility of *C. lytica* as a self-printing, photonic ink is established by this study. In summary, autonomous surface assembly of *C. lytica* under ambient conditions and across multiple length scales circumvent challenges that currently hinder production of ordered materials in industrial settings.

## Introduction

Fluctuating and harsh environments challenge biological systems to evolve mitigating strategies for survival. Structural hierarchy is a prevalent response to such challenges and is the basis of conspicuous material properties and functionality^1,2^. Though researchers have developed biomimetic materials that incorporate nature’s design principles, manufacturing hierarchical materials at industrial scales remains difficult^3–5^. Biomanufacturing has the potential to reduce energy consumption and carbon emissions by using living organisms to produce complex materials^6–8^. A particular area of focus are bacterial inks where embedded cells enable 3D printing of functional materials^9,10^. Researchers report laboratory successes in patterning bacteria for additional control by using nanostructures, electric fields and optogenetics^11–13^.

Structural coloration, an emergent property linked to bacterial colonies, derives from the interaction of light with recurring, hierarchical, submicron structures. In multi-cellular organisms, structural color enhances essential functions including light harvesting, mating, defense, and communication^14–17^. Iridescence, or angle-dependent structural color, often involves combinations of pigments, iridophores and multi-layered structures which are attached to membranes in eukaryotes^18,19^. Prokaryotic systems including genera *Cytophaga*, *Flavobacterium* and *Cellulophaga* also display iridescence and the precise mechanisms involved are currently under study by several groups^20–22^. Iridescence by these bacteria is defined as structural color with an angle-dependent peak intensity. Note that this is distinct from the iridescence generally associated with butterflies and mollusk shells where the reflected wavelength is also angle-dependent. Under laboratory conditions, strains of *Cellulophaga lytica* self-organize into cooperative 3D communities, known as biofilms, generating iridescence of discrete wavelengths^22,23^. Coordinated gliding motility facilitates short-range ordering of bacteria over large areas^24–26^. In studies by Kientz et al., glitter-like iridescence was generated by various strains of *C. lytica,* including marine isolates DSM 2040 and CECT 8139 from seawater aquaria in La Jolla, USA and Oleron Island, France, respectively^23^. The ability to glide and environmental factors such as temperature, salinity, etc. were shown to impact iridescence^20,27,28^. *C. lytica* DSM 7489 (aka CIP 103822 and Lim 21T; originally isolated from beach mud in Limon, Costa Rica) was significantly less iridescent and used as a negative control in their experiments^20,22,23^. Pursuant to our goal to develop hierarchical materials which are amenable to biomanufacturing, this study characterized iridescent biofilms of the commercially available strain of *C. lytica*, DSM 7489 and developed strategies for controlling biofilm optical and spatial properties. Thus, the utility of *C. lytica* biofilms as a platform for developing sustainably manufactured ordered materials was demonstrated.

## Results

### Biofilm properties

**Figure 1:**
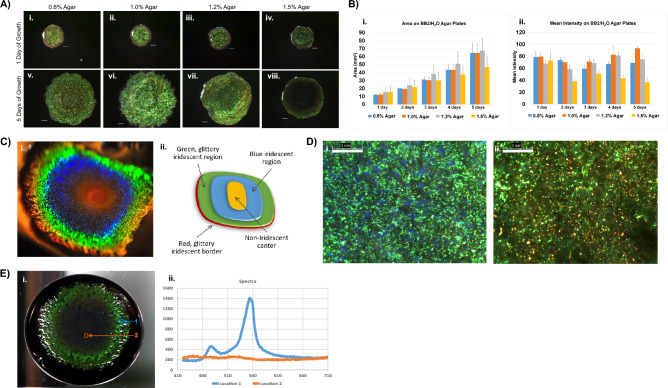
Given particular growth conditions, *C. lytica* 7489 makes intensely iridescent biofilms though previously reported as lacking color. (**A**) Representative *C. lytica* 7489 biofilms were grown at 27°C on BB2/H_2_O agar plates containing 0.8%, 1.0%, 1.2% or 1.5% agar. Biofilms were imaged each day for 5 days. Agar concentration impacts color saturation and expansion of DSM 7489 *C. lytica* biofilms. Scale bar = 1 mm. (**B**) Areas (i) and mean intensity (ii) measurements of biofilms in A were recorded daily using ImageJ. Data are presented as averages for each timepoint with standard deviation shown as ±error (n = 10 for each timepoint). (**C**) *C. lytica* 7489 is capable of generating a range of intense colors as shown in this photo of a biofilm acquired from an oblique viewing angle (i) and schematic showing the concentric coloration (ii). (**D**) Optical images showing that regions of the biofilm appearing green (i) and red (ii) macroscopically are mosaics of pointillistic colors. (bar = 1 mm) (**E**) A representative hyperspectral data cube reveals region-specific variations in signal intensity in mature biofilms. (i) The biofilm was grown in a 10 cm petri dish on nutrient agar containing black ink. Note that the detector is normal to the surface of the biofilm. Its position is the reason for the reduction in reflection intensity compared to the biofilm in (**C**-i). Outer regions of the biofilm generate an especially sharp peak centered near 550 nm suggesting constructive and coherent reflection through the biofilm. (ii, location 1) In contrast, reflections in the center region remain near baseline. (ii, location 2)

*Cellulophaga lytica* 7489 biofilms grown for 24 h on BB2/H_2_O media containing various agar concentrations were compared (Fig. 1A, B). Color patterns differed after 5 days with the 1.0% agar supporting the brighter colonies. Culture media containing greater than 1.2% agar permitted growth, though spreading of the colonies was constrained compared to those containing 1% agar or less. Biofilms from 1.5% agar were smaller with lower mean pixel intensity. Unless specifically noted, remaining data in this study was based on biofilms from 1% agar plates containing BB2/H_2_O. Continued incubation on 1% agar resulted in biofilms having concentric, gradient coloration (Fig. 1C). Though the relative dimensions and intensities of these regions varied among samples, the order of coloration in mature biofilms did not change. In addition, the biofilm centers often turned a golden-orange color, possibly due to the pigment zeaxanthin which is known to be present in *C. lytica*^29^. Progressing radially outward, the next bands of color were blue followed by a glittery green, and a narrow band of red around the perimeter of the biofilms. Magnification revealed that the colors are mosaics where the predominant color in these millimeter domains determined the perceived macroscopic color (Fig. 1D). Importantly, the mosaics show the potential of *C. lytica* biofilms to reflect a range of colors.

A Resonon benchtop hyperspectral system was used to generate data cubes representing bulk optical responses of living *C. lytica* biofilms (Fig. 1E). Spectra spanning the visible range were collected and show increased intensities centered near 550 nm. The sharp peak suggests that constructive and coherent reflections occur through the biofilm. In contrast, intensities associated with the center of the biofilm remain near baseline levels. In other experiments, using backscattered geometry and variable excitation angles, a dependence on detection angle was established (Supplemental Fig. 1).

### Cellular arrangement, packing and morphology within *C. lytica* 7489 biofilms

**Figure 2:**
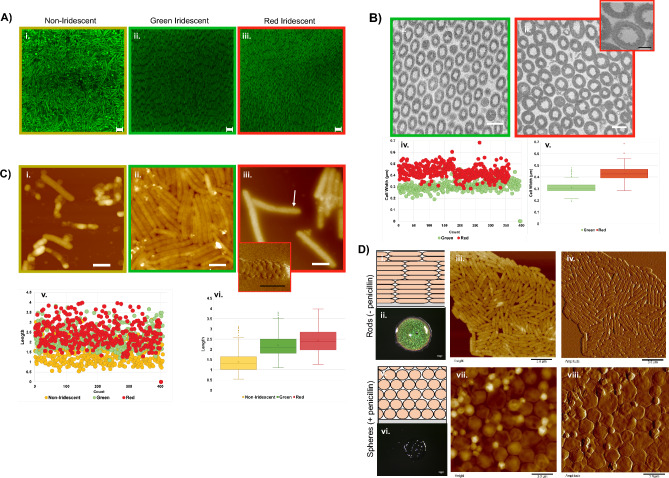
Microscopy of biofilm cells. (**A**) Confocal images of biofilms stained with SYTO9 showing that cellular organization differs between non-iridescent (i) and iridescent regions (ii, iii) (bar = 2.0 µm). (**B**) Transmission Electron Microscopy (TEM) cross section images of green (i) and red regions (ii). (bar = 0.5 µm) Inset (iii) showing small protrusions surrounding the cell walls (bar = 200 nm). Width measurements (iv, v) also differ by region. (**C**) Atomic Force Microscopy (AFM) height images of non-iridescent (i), green iridescent (ii), and red iridescent (iii) regions showing that distinct cellular morphologies are associated with each region (bar = 1.0 µm). Inset (iv) is an AFM amplitude image of the region indicated by the arrow (bar = 0.5 µm). Length measurements from specified biofilm regions (v, vi). (**D**) 2-day old biofilms grown in ambient conditions on BB2/H_2_O agar (optical scale bars = 1 mm; AFM bars = 3 µm). Schematic showing typical arrangement of cells in iridescent biofilms (i). Optical image of iridescent biofilm. (ii) AFM height (iii) and amplitude (iv) images of cells from iridescent biofilm showing typical rod shape morphology. Schematic showing predicted arrangement of cells in biofilms grown when sublethal penicillin is added (v). Optical image of biofilm showing that sub-lethal penicillin disrupts structural coloration. (vi) AFM height (vii) and amplitude (viii) images of cells confirm conversion to spheres due to penicillin treatment.

Previous studies of *Flavobacterium* biofilms revealed that cellular spacing and morphology impacted reflected wavelengths^21,30^. In order to understand color differences in *C. lytica* 7489 biofilms, complementary microscopy techniques were applied. The arrangement of *C. lytica* within biofilms was visualized using confocal microscopy of fixed biofilms stained with SYTO9 (Fig. 2A). Regions of the biofilm not associated with iridescence contain randomly oriented cells, though small clusters of aligned cells were also observed. Spherical structures, also present in this region, increased in number as the imaging plane approached the substrate (Supplemental Fig. 2A). In contrast, cells in iridescent regions were closely packed and ordered into planar, polycrystalline layers as confirmed in the fast Fourier transform (FFT) of the image of the green iridescent region (Supplemental Fig. 2B). Ordering persisted over tens of microns with grain boundaries indicated by a change in the directions of the cells.

Eukaryotes embed components contributing to iridescence in tissues that can be handled and manipulated during study. *C. lytica* iridescence however, is derived from independent, loosely associated cells which are easily separated by mechanical disturbances, including turbulence from excessive hydration. Crosslinking the cells with glutaraldehyde preserved the iridescence and allowed removal of intact biofilms from the agar for characterization by transmission electron microscopy (TEM). Cross sectional electron micrographs of fixed *C. lytica* 7489 biofilms confirmed the periodicity in iridescent areas (Fig. 2B). Cells in the green and red regions had mean widths of 310 nm and 428 nm, respectively indicating that changes in cell width contributed to color variations. Also, higher magnification TEM images of cells from the red region revealed small protrusions surrounding the cell walls (Fig. 2B-iii). Lateral images of biofilms, acquired using scanning electron microscopy (SEM) show that ordering occurs through the full thickness of the biofilms which ranges from 15 to 60 µm (Supplemental Fig. 2C).   

Given the differences in width observed in the TEM data and the association with specific regions of the biofilm, we sought to compare the morphologies of cells using atomic force microscopy (AFM) (Fig. 2C). Cells from the non-iridescent region were irregular, associated with spherical protrusions and often appeared deflated. In contrast, cells from both the red and green iridescent regions maintained regular morphologies and dimensions, as expected with healthy rod-shaped bacteria. Despite repeated attempts to disrupt aggregates, cells from green iridescent regions remained closely associated, indicating that cell surface properties also varied by region (Fig. 2C-ii). In stark contrast, cells from the red region were often covered in membrane vesicles (Figs. 2C-iii and iv and Supplemental Fig. 3). Gram-negative bacteria including *C. lytica* are known to generate outer membrane vesicles^31,32^. However, the complete surface coverage by the vesicles was striking. The better-defined AFM images suggested that membrane protrusions observed in the TEM cross-sections were vesicles. A comparison of the lengths of the cells from the different regions revealed that non-iridescent cells are shorter than those in the green or red regions with mean values of 1.39 µm, 2.20 µm and 2.42 µm, respectively (Fig. 2C-v, vi). Taken together, the imaging data indicated distinct size and surface topologies depending on the location of the cells within the biofilm.

Peptidoglycan in Gram-negative bacteria is a cage-like polymer that provides the cell’s mechanical stability. Located between the cytoplasmic membrane and the outer membrane, its shape determines the morphology of the bacteria. Lysozyme and beta lactam antibiotics are widely known to disrupt the peptidoglycan and result in cell lysis. At sub-lethal doses however, Gram-negative bacteria including *E. coli* survive the treatment but are converted from rods to spheres^33–35^. To determine whether coloration requires intact peptidoglycan, 30 µg/mL penicillin was added to the agar plates before inoculation with *C. lytica* (Fig. 2D). After 48 h, treated biofilms lacked iridescence due to the conversion of *C. lytica* into spheres, indicating the importance of the peptidoglycan to iridescence. These results also showed that exogenous shape modifying reagents can be used to impact biofilm optical properties.

### Scalability and tunability of biofilms for manufacturing

**Figure 3:**
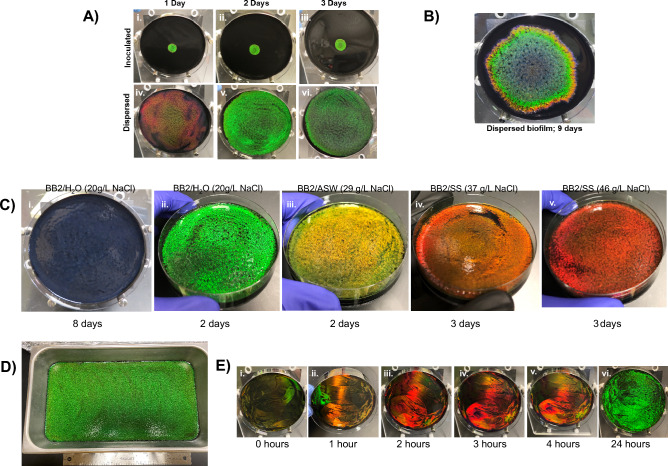
Monochrome biofilms. (**A**) A comparison of inoculated (i–iii) versus dispersed (iv–vi) biofilms at ambient temperature. Dispersed biofilms peak in monochrome color and fill the plate at 2 days (v). (**B**) Whereas, inoculated biofilms develop banded coloration and require significantly more time to cover the surface. (**C**) Photos showing that dispersing *C. lytica* cells leads to monochrome biofilms of various colors presumably by simultaneously exposing all the cells to homogeneous growth conditions (nutrients, metabolites, etc.). The range of colors can be accessed by extending the growth period (i) or changing media salinity (ii – v) using sea water simulants. Effective NaCl in recipe given in parenthesis. Plates were grown in ambient conditions for the indicated time. (**D**) A large green biofilm was generated in 3 days in ambient conditions by dispersing a proportional inoculum on the surface of a 41 x 23 cm pan. (**E**) Sequential application and dispersal of 50-fold concentrated aliquots of culture reduces monochrome biofilm formation to 24 h or less, suggesting that the cells immediately begin to organize and that cell density will be an important consideration for industrial manufacturing applications. Plates were incubated at 27 °C between cell applications. Additional trials shown in Supplemental figure 4E,F.

*Cellulophaga lytica* biofilms expanded radially from the point of inoculation and displayed banded colors (Fig. 1A). This patterning was likely a consequence of temporal differences in cell morphologies induced by changes in the local environment (e.g. nutrient availability, pH, metabolites, etc.) during cell growth. Since monochrome biofilms would indicate consistent morphologies, we reasoned that adding sufficient cells at once to the entire agar surface would expose all the cells to the same environment simultaneously and synchronize the reflected color. This hypothesis was tested by comparing biofilms initiated with a localized 100µL inoculum to those where the same volume inoculum was dispersed over the agar using a cell spreader. (Fig. 3A) Representative photographs show that after 1 day of growth in ambient conditions, inoculated biofilms reflected bright green iridescence around the center of the plate whereas dispersed biofilms were diffusely red across the plate surface. After 2 days of growth, both preparations yielded bright green iridescence though dispersed biofilms covered the entire surface of the agar. Iridescence in dispersed biofilms waned considerably after 3 days. With additional time, inoculated biofilms expanded to cover most of the agar surface (Fig. 3B). Therefore, dispersing the cells both shortened the time to optimal iridescence and expanded the area of the plate covered with ordered monochromatic bacteria.

Since Kientz et al. showed that salinity impacts coloration of CECT 8139 biofilms, DSM 7489 biofilms were similarly tested^27^ (Fig. 3C). The media was augmented with either Instant Ocean (BB2/ASW) or Lake Products Sea Salt ASTM D1141-98 (BB2/SS) sea salt analog. Compared to the dispersed biofilms on BB2/H_2_O, growth on BB2/ASW and BB2/SS generated nearly monochrome colonies with red shifts correlating to the amount of the sea salt analog added to the media. Given that the analogs differ mainly in NaCl concentrations, we tested whether increasing the concentration of NaCl is sufficient to generate the red shifted colors by growing biofilms on BB2/H_2_O plates supplemented with varying amounts of NaCl (Supplemental Fig. 4A). Increasing concentrations of NaCl in localized plates led to red shifted colors, thus allowing the properties of the biofilm to be manipulated exogenously. The bands shown in Fig. 1 reflect the *range* of colors that can be generated by *C. lytica* biofilms. Here we show that that red, an early color on BB2/H_2_O agar plates, was generated by increasing the salinity of the growth media whereas blue was generated by extending the period of growth. Taken together, we conclude that the temporal aspect of coloration is derived from cellular responses to the local environment.

Dispersing a proportionally larger inoculum across a 41 cm × 23 cm agar surface generated a surface-filling monochrome biofilm after 3 days of incubation at room temperature, whereas a localized inoculum led to a restricted iridescent area (Figs. 3D, Supplemental 4B). To further reduce the time for generating large area monochrome biofilms, we tested whether increasing the cells in the inoculum leads to faster iridescence. Specifically, overnight cultures were concentrated before dispersing the cells on the agar. The speed with which iridescence appeared correlated with the fold concentration of the inoculum dispersed across the surface (Supplemental Fig. 4C). A layering strategy further accelerated iridescence formation where 50-fold concentrated cells were serially applied to the agar plates (Figs. 3E, Supplemental Figs. 4E, 4F, 5, 6) Notably, vibrant yellow and red reflections appeared early, consistent with the sequence of coloring in the inoculated biofilms where red is transiently seen in periphery of the inoculated plate followed by green. At 24 h, the entire plate reflected green. Together, these results show that cells are able to organize quickly into photonic structures and that cell number is a limiting factor for the formation of iridescent biofilms.

### Incorporating biofilms into materials

**Figure 4:**
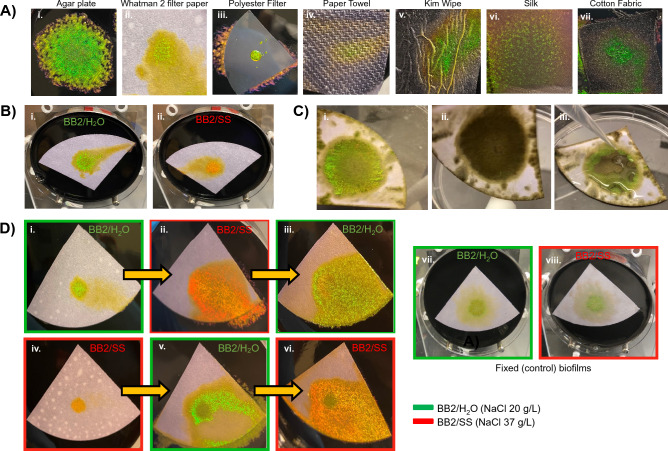
Ambient growth of iridescent biofilms on porous substrates including paper facilitates handling for characterization and downstream processing. (**A**) *C. lytica* biofilms were grown in ambient conditions on a variety of porous substrates. (**B**) Whatman 2 filter paper placed atop nutrient agar is one of several porous substrates that allow *C. lytica* to form iridescent colonies. As on agar plates, living paper-associated biofilms (PABs) are green after 3 days of growth atop BB2/H_2_O agar (A-i) and red-shifted when salinity increases as on BB2/SS (A-ii). (**C**) PABs retain their iridescence after removal from agar and fixation with glutaraldehyde (B-i). Drying the fixed PABs with nitrogen causes them to lose their iridescence (B-ii). However, the structural color is restored upon rehydration (B-iii). (**D**) Living PABs retain their ability to respond to environmental cues. PABs from BB2/H_2_O agar reflect mostly green until they are moved to BB2/SS plates where reflections are shifted red (C-i and C-ii, respectively). Similarly, PABs originating on BB2/SS agar plates are red but change to green when placed on BB2/H_2_O (C-iv and C-v, respectively). In both cases, biofilms are able to revert back to original color when returned to the original media condition (C-iii and C-vi). Fixed biofilms do not show this dynamic behavior (C-vii and C-viii).

Growth on porous substrates including qualitative filter paper (Whatman, Grade 2), polyester track-etch membranes (Sterlitech), cotton fabric and silk atop nutrient agar was tested. Media retention and nutrient diffusion allowed biofilm growth and expansion on these substrates. Importantly, reflected color patterns were similar to those of biofilms grown directly on the agar plates (Fig. 4). Of the tested substrates, filter paper was selected for further study due to the quality of the iridescent biofilm, low substrate cost and flexibility. After growth, paper-associated biofilms (PABs) were transferred to agar containing glutaraldehyde for fixation to preserve iridescence. The paper facilitated handling of the biofilm during characterization and subsequent processing. Iridescence of the PABs was lost after drying, but was quickly restored with the addition of water (Fig. 4C). PABs tolerated multiple cycles of drying and rehydration with minimal loss of iridescence. AFM imaging of the fixed paper biofilms confirmed the crystalline arrangement of cells (Supplemental Fig. 7B).

**Figure 5:**
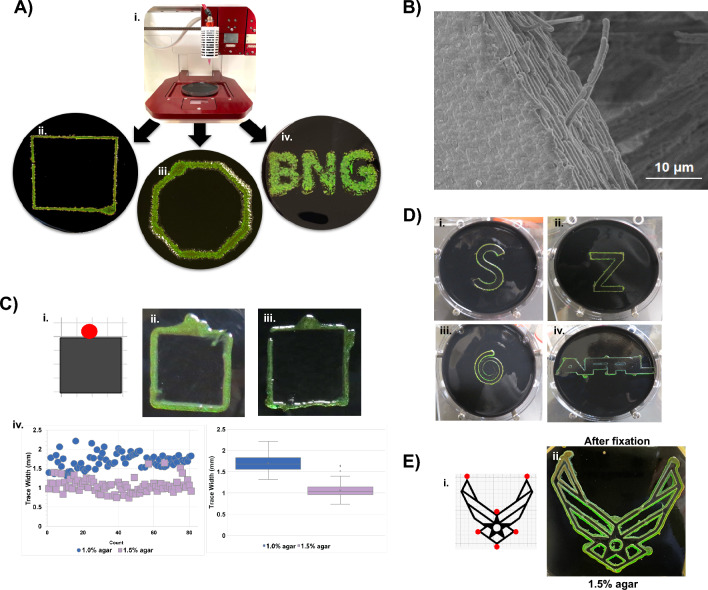
*C. lytica* can be used as an iridescent bioink. (**A**) 3D printed designs containing *C. lytica* were generated on agar using an Allevi 3 Bioprinter setup (A-i to A-iv). As previously shown for dispersed biofilms, increased salinity red shifts the *C. lytica* ink’s reflection. (Supplemental fig. 9) (**B**) SEM image of edge of 3D printed biofilm showing ordered cells of the printed biofilm. (**C**, **D**) *C. lytica* trace the edges of a paper template (e.g. schematic in C-i). The red circle on the template design indicates the site of inoculation. Cells behave like a self-printing bioink to write various patterns in ambient conditions. (B) Increasing the agar concentration from 1.0% (C-ii) to 1.5% (C-iii) reduces the width of the tracing as revealed in Keyence measurements (C-iv). This result suggests that gliding motility is modulated in a way that confines the cells closer to the template on the higher concentration agar. (**D**) Additional tracings show BACTracing can be used with shapes of varying complexity, angles and connections. Agar concentration can be used to confine the traces when the distance between features is small as is the case in intricate patterns such as the Air Force Symbol (supplemental Fig. 6c). (**E**) Template of a complex pattern (i) and its BACTraced counterpart (ii) after fixation showing that the iridescent pattern can be preserved.

Living PABs (i.e. without fixation) retained their ability to respond to environmental cues. For example, PABs on BB2/H_2_O agar plates reflected mostly green until they were moved to BB2/SS plates where reflection was shifted red and vice versa (Fig. 4D). In both cases, biofilms reverted back to their original color when returned to the original media condition (Supplemental Fig. 8A). This reversion did not occur when cells were fixed with glutaraldehyde, confirming that the living cells’ ability to sense and respond was required for the color changes. As with the localized and dispersed biofilms, exogenous reagents such as penicillin and lysozyme can be delivered to the biofilm through the paper substrate to control biofilm properties (Supplemental Fig. 8B).

### *Cellulophaga lytica* biofilms as iridescent inks

Seeking to develop ordered bacterial inks based on *C. lytica*, an Allevi 3™ bioprinter was employed to deposit the cells in pre-programmed patterns and Google SketchUp to create STL design files ranging from shapes to letters. Printed cultures maintained the desired patterns on BB2/H_2_O plates after incubation and growth at 27 °C (Fig. 5A). The patterns were also printed on BB2/ASW agar plates where increased salinity again red shifted the color. (Supplemental Fig. 9A) Subsequent SEM images of the crosslinked biofilms showed that the bacteria were closely packed and ordered in the printed shapes (Fig. 5B).

Motility in bacteriodetes is required for generating iridescent biofilms^20,30^. Confocal images from this study show alignment between cells that likely result from a directional flow of cells on the agar surface combined with interface interactions. We hypothesized that obstacles in the path of gliding cells would alter the direction of cell flow and that this obstructive steering could result in a type of “self-printing” bacterial ink. To test this idea, paper templates were placed on nutrient agar plates before adding an inoculum adjacent to the paper (Figs. 5C, Supplemental Fig. 9B) During incubation, the cells generated iridescent traces along the edges of the templates via a process we call bacterial autonomous collective tracing (aka “BACTracing”). BACTracing occurred on both on 1% and 1.5% agar plates, however, the higher agar concentration led to narrower traces. Temperature also impacts the quality of patterns generated with BACTracing (Supplemental Fig. 10). Increasingly complex patterns were printed using a small number of inoculation sites suggesting that BACTracing can be a facile approach for advanced printing of living material (Fig. 5E).

## Discussion

The crystalline self-assembly and robust growth of *C. lytica* in ambient conditions, across multiple length scales, could be exploited to circumvent challenges currently hindering replication of ordered materials in industrial settings. Importantly, the genetic tractability of prokaryotic systems makes them amenable to tailoring using synthetic biology. Since the bacterium is the structural unit, it is possible to generate dynamic or reconfigurable iridescence by controlling the behavior and dimensions of a single cell type. These advantages make bacteria that generate structural colors candidates for large scale, affordable, ‘green’ and field-friendly optical materials as well as tunable templates for ordered materials. We identified conditions that cause *C. lytica* DSM 7489 to form brilliant biofilms, though this strain was previously determined to lack intense iridescence^20,22,23^. Imaging data in the current study indicated that changes in color reflected from these biofilms were attributed to variations in morphology and that *C. lytica* OMVs may play a role in color selection by expanding the effective cell width (Supplemental Fig. 11). Schertel et al. determined that color changes derived from variations in lattice constants, due to differences in intercellular spacing or cellular dimension^21^. Johansen et al. confirmed that transposon mutants of *F. Johnsoniae* developed biofilms reflecting different colors because of altered cellular dimensions^30^. Quantifying the spacing in biofilms is challenging because the dehydration required for cross-sectional electron microscopy may alter the natural spacing between cells and optical measurements lack the resolution to measure intercellular spacing. Our approach to assess the unit cell dimensions directly with single-cell microscopy revealed that biofilm constituents transiently modulate cell widths in response to their local environment. Importantly, genetic modifications are not responsible for these phenotypic changes as was the case in previous studies. We conclude that biofilm color variations derive from changes in morphology and cell width.

To our knowledge, this study represents the first attempt at incorporating *C. lytica* biofilms into materials and highlights qualities that are advantageous for manufacturing biotemplated materials at industrial scales. Their self-assembly at ambient temperatures avoids complex fabrication techniques, and potentially lowers heating costs. Rapid generation of *C. lytica* biofilms beyond laboratory scale dimensions is achievable through simple changes in the way the cells are applied to the agar. Whereas maintaining the diffusion of nutrients, oxygen and metabolites is a major challenge in large fermentation processes, biofilm growth conditions are easily maintained and agar constituents can augment properties. We also show that growing biofilms on porous substrates allows them to be handled during transfers and processing. All together, these results confirm that uniform biofilms can be generated in ambient conditions, at useful scale and within practical timeframes. These are important considerations for biomanufacturing materials based on *C. lytica*.

The utility of *C. lytica* as bacterial ink is also demonstrated in this study. The gliding motility of *C. lytica* enable bioprinting of patterns on agar surfaces and outline templates via BACTracing. Since trace properties are tunable by the growth conditions, self-printing complex designs with fidelity is possible. In principle, these bioprinting processes can be used to generate large-scale designs for a variety of applications, and future studies will be aimed at exploiting the chemical diversity available on the cell surface for the development of novel materials. As living materials, engineered biofilms can be used as sensing platforms which visibly report conditions based on color. An area of special interest for the future is the development of genetic parts for manipulating iridescent bacteria including *C. lytica.* Such tools are needed to enable synthetic biology approaches that would expand functionality of the biofilms as a versatile platform.

## Materials and methods

### Bacterial cultures

All experiments in this paper were conducted with *Cellulophaga lytica* DSM 7489 which were purchased from Leibniz Institute DSMZ-German Collection of Microorganisms and Cell Cultures GmbH. Glycerol stocks were made from the cells using BB2/H_2_O, a modified marine media^36^. Per liter, BB2/H_2_O contains Difco marine broth 2216 (18.7 g), tryptone (5.0 g), yeast extract (2.5 g), sodium chloride (10.0 g) pH 7.8. Note that this recipe differs from the marine agar recipe used in the Kientz studies with the substitution of LB components for some of the marine broth. Where specifically noted, culture media was augmented with commercially available sea salt analogs, either (30–45 g/L) Instant Ocean (BB2/ASW) or Lake Products Sea Salt ASTM D1141-98 (BB2/SS). Biofilms grown on the standard BB2/H_2_O media were included as control samples in experiments examining the effects of salinity on growth and color. Formulations of the marine broth and the Lake Products ASTM D1141-98 are available from the manufacturer, making it possible to estimate the NaCl content in BB2/H_2_O and BB2/SS to be around 20 g/L and 37 g/L, respectively. Because the makeup of the Instant Ocean is proprietary, the composition is not available from the manufacturer. However, researchers have used analytical methods to determine the composition and based on their results we estimate the NaCl concentration in BB2/ASW to be around 29 g/L^37^. Liquid cultures were shaken at 200 rpm and grown at 27 °C.

### Biofilm growth

Overnight cultures of *C. lytica* 7489 were diluted with BB2/H_2_O or concentrated as needed via centrifugation to achieve an OD_600_ equal to 1. Poured 10 cm culture plates contained media, 0.8% to 1.5% agar and supplements (e.g. salts, penicillin, etc.) as specified in the text. Plates at the lower end of the range proved to be better substrates for iridescent biofilm expansion and 1.0% agar was used for routine growth. This determination was made after comparing the size and intensity of biofilms grown on various agar concentrations (Fig. 1). In most cases, 1% black ink (Higgins Fountain Pen India Black) was added to the agar solution to provide contrast so that the natural iridescence of the biofilm could be more easily observed. Typically, 10 cm agar plates were inoculated using 100 μL of liquid culture. In order to make “dispersed” plates, 100–400 µL of cell culture was applied to the center of the culture plate before subsequently using a cell spreader to disperse cells across the agar surface. When making a direct comparison between inoculated and dispersed biofilms, 100 µl of cell culture was used. Biofilm formation was accelerated by concentrating the overnight culture as indicated and sequentially dispersing aliquots of the concentrated cell suspension. These applications were repeated with 5 min intervals between applications to allow the media to absorb into the agar. In order to grow biofilms on paper, swatches of autoclaved Whatman Grade 2, qualitative cellulose filter paper (containing ~ 8 µm pores) were placed atop nutrient agar culture plates before inoculation. Incubation temperatures for the biofilms ranged from ambient to 27 °C. Other porous substrates were similarly prepared. At defined intervals, biofilms were imaged using a Leica, EZ4 HD microscope. Using ImageJ software, calibrated images were analyzed to determine the area of each biofilm and its mean pixel intensity. Separately, biofilms were also photographed using a Canon PowerShot SX40 HS camera. In most cases, photographs were acquired after placing the culture plate on an augmented adjustable angle mounting plate as described in the supplemental Fig. 12.

### Biofilm fixation

Agar surrounding the biofilm was excised using a spatula and replaced with 1–2% molten agar supplemented with 0.5–2% glutaraldehyde or 4% paraformaldehyde. Biofilms were stored overnight to allow the glutaraldehyde fixative to diffuse through the biofilm. Paper biofilms were transferred to a dollop of 1–2% agar containing 0.5–2% glutaraldehyde for fixation. These preparations were important because they facilitated further characterization of the biofilms and preserved the ordered arrangement of the bacteria.

### Spectrometry measurements

Data cubes of the bulk optical responses of living *C. lytica* biofilms were generated using a Resonon benchtop hyperspectral system. The PikaXC2camera is a line scan, push broom imager where each frame is a single line of data and a full two dimensional image is produced by linearly translating the sample under the camera and stacking the data line by line. In the configuration used for these experiments, the PikaXC2 camera was anchored in a fixed position, perpendicular to the sample. Prior to acquiring data, the camera was calibrated to remove dark noise from the data. A white calibration was also performed in order to account for illumination and camera response. Using concentric circles, stage speed and frame rate were adjusted until they were synchronized and produced an image free of distortion. Data cubes were collected in reflectance mode at 27 Hz. Data was analyzed using Spectronon, a hyperspectral data visualization and analysis software package.

### AFM microscopy

In order to examine discrete populations of the biofilm, sterile inoculating loops were used to collect cells from specific region of the biofilm. Cells were subsequently dispersed into media containing 0.25% glutaraldehyde. After incubating the sample on a rocker for 10 min, the fixed cells were pelleted via centrifugation and washed. A subsequent centrifugation allowed the cells to be collected and concentrated in a smaller volume. After applying 5 µl of the glutaraldehyde-fixed suspension to freshly cleaved mica and allowing it to dry, the sample could be loaded into the microscope for AFM imaging. This approach was especially important for assessing the morphology of the cells that make up the thin red border. The ‘pick-up’ method was also employed to transfer cells from the biofilm to mica or gelatin-coated mica. Basically, the substrate (glass slide, plain mica, gelatin-coated mica) was inverted onto the biofilm ensuring good contact and removed. Iridescent and non-iridescent regions were marked with a felt pen on the back of the substrates. After lifting the substrate, 200 µl of 0.25% glutaraldehyde was added to the sample for 20 min. Loosely attached cells were removed by vigorously washing the sample in a stream of water. After drying, a layer of fixed cells remains for imaging in air or liquid. Where used, gelatin-coated substrates were prepared as previously described by Doktycz et al.^38^.

In order to prepare for AFM imaging, PABs were removed from the agar plate and dried. The paper edges were weighted down during drying to prevent curling. Drying was achieved overnight in ambient conditions or accelerated with a stream of dry nitrogen. The dried samples were imaged directly in air. AFM was performed either using a Bruker Icon in tapping mode or a Keysight 9500 AFM in acoustic mode in ambient conditions. Silicon cantilevers (Nanosensors PPP-NCHR) having nominal spring constants and resonance frequencies of ~ 42 N/m and 330 kHz, respectively were used for imaging in air. Alternatively, liquid imaging was performed using a Keysight 9500 AFM in TopMac^®^ mode with MacLever type II cantilevers having a nominal spring constant 2.8 N/m and ~ 30 kHz resonant frequency in water. Scan rates ranged from 0.5 to 2.0 Hz with 512 data points per line.

### Transmission electron microscopy

After fixation, discrete regions of the biofilms were transferred to a 90 °C water bath with gentle agitation. Though the hot water causes the agar to disintegrate, the fixed biofilm maintains its integrity and floats freely in the water as flakes. Once separated, the biofilms are stable in water and retain the distinct iridescence (Supplemental Fig. 8). Glutaraldehyde-fixed *C. lytica* films were placed on polymerized low melt agarose (2%) before covering with molten agarose. Fixation continued in osmium tetroxide overnight. Fixation in Osmium tetroxide followed a protocol of a 2 h fixation and then three 15 min washes in a Hepes buffer wash. After washing, dehydration in a series of alcohols for 15 min each (10%, 20%, 30%, 40%, 50%, 60%, 70%, 80%. 90% and three changes of 100% ethanol). Epon resin was used to embed the samples. First the samples are put into resin at 1:3 (one part resin to 3 parts 100% ethanol for 2 h. Then 1:2 for 2 h and then 1:1 overnight. The next morning a fresh change into 100% resin for an hour and then using fresh resin embedded into flat silicone embedding molds. These samples are placed in a 60 °C oven overnight to polymerize. Using an Ultramicrotome with a 35° DiaTome diamond knife sections of 70 nm thickness were collected onto Cu mesh grids. After drying, the grids were vapor stained using RuO4. TEM imaging was done at 200 kV using an FEI CM200. ImageJ was used to measure and compare the widths of green and red cells from the TEM cross sections.

### Confocal microscopy

Paraformaldehyde-fixed biofilms were separated from the agar and submerged in a solution of SYTO9, a membrane permeable DNA dye, for 20 min. SYTO9. After collecting the biofilms on glass slides, they were imaged using a Zeiss LSM 700 upright confocal microscope outfitted with a 63× objective.

### 3D bioprinting

An Allevi 3 Bioprinter was used to dispense *C. lytica* cells onto black ink nutrient agar plates. Google SketchUp was used to create STL files of the designs which were then uploaded into the web-based Allevi 3 Bioprinter software. The air compressor was turned on to allow the pressure to build to around 125 PSI. The liquid culture was loaded into a 5 mL syringe, inserted into one of the extruders, and locked into position. The other two extruders were detached and placed to the side. Next, autocalibration was performed before an agar plate was placed on the build table and the extruder was manually calibrated to the correct X, Y, Z origin. Printing parameters such as printing speed and pressure were adjusted in order to dispense a steady stream of culture with minimal pooling on the agar surface. The optimal print speed was determined to be 2 mm/s with a pressure of 3.5 PSI. After printing, the syringe and needle were soaked in 70% ethanol to sterilize them for future print jobs. The resulting plates were incubated at 27 °C and observed every 24 h for bacterial growth.

### BACTracing

Paper templates were cut from Whatman Grade 2, qualitative cellulose filter paper using a Cricut Explore One cutting machine. Designs were created in the provided software. Cuts were made using a standard replacement blade on either a light or standard mat. In order to compare the performance of *C. lytica* on 1.0 and 1.5% BB2/H_2_O agar plates, autoclaved templates were placed atop the agar before inoculating them at select locations with 3 µl of overnight cell culture. Cells were incubated and photographed at defined intervals. The images and measurements in Fig. 5 were acquired after 4 days of growth at ambient temperatures.

### Keyence measurements data analysis

After removing the paper templates, biotraced squares were excised from the agar and transferred to a silicon wafer (without fixation). After placing the sample on the Keyence 3D laser scanning confocal microscope (Profile Measurement VK-X200 series), a 5× objective was used to collect images from 3 non-overlapping regions of each side of the square, excluding the side containing the inoculation site. Each field of view was subdivided into approximately three equal regions. A cross sectional measurement was made in each region of the combined optical/laser image, yielding a total of 27 measurements per square. The Multifile analysis module provided with the Keyence system was used for image processing.

### Supplementary Information

Supplementary Figures.

Supplementary Video 1.

Supplementary Video 2.

Supplementary Video 3.

## Data Availability

The datasets used and/or analyzed during the current study are available from the corresponding author on reasonable request.
