# Supplementary material for: Iridescent biofilms of Cellulophaga lytica are tunable platforms for scalable, ordered materials
Source: Sci Rep. 2023 Aug 14;13:13192. doi: 10.1038/s41598-023-38797-0 (PMC10425352; doi:10.1038/s41598-023-38797-0)
Supplement: Supplementary file 1 — Supplementary Figures. [file 41598_2023_38797_MOESM1_ESM.pdf]

Iridescent Biofilms of *Cellulophaga lytica* are Tunable Platforms for Scalable, Ordered Materials

*Claretta J. Sullivan[1], Kennedy Brown[1], Chia Hung[1], Joseph Kuo-Hsiang Tang[1], Mark DeSimone[1,2], Vincent Chen[1], Pam Lloyd[1], Maneesh Gupta[1], Abby Juhl[1], Wendy Crookes-Goodson[1], Milana Vasudev[1,2], Patrick Dennis[1], Nancy Kelley-Loughnane[1]

[1] Materials and Manufacturing Directorate, Air Force Research Laboratory, WPAFB, OH 45433 U.S.A.
[2] Department of Bioengineering, University of Massachusetts Dartmouth, Dartmouth, MA 02747 U.S.A.

Supplemental Information:
Note: The datasets used and/or analyzed during the current study are available from the corresponding author on reasonable request.

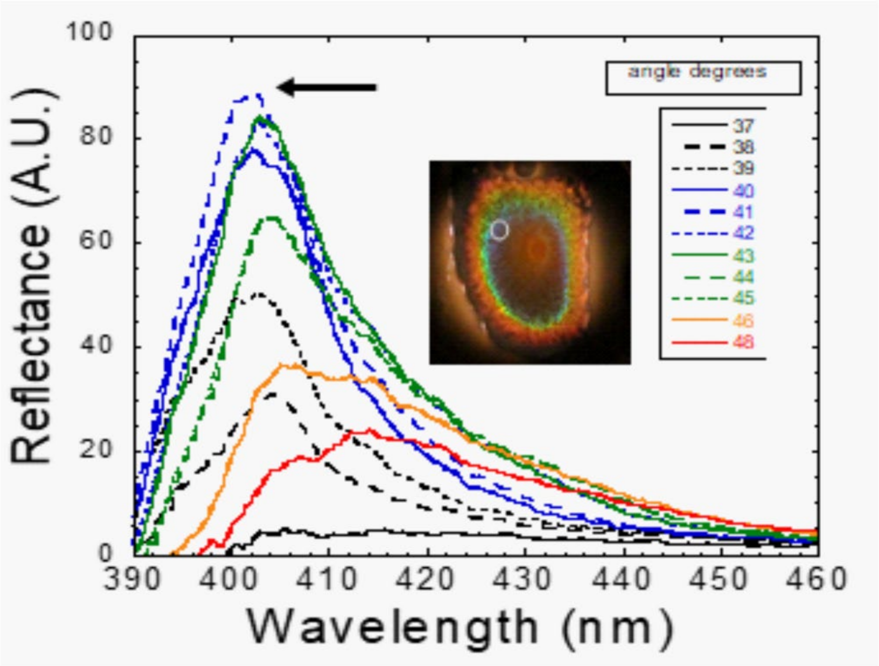

Supplemental Figure 1. Spectrometry Measurements. An *ad hoc* spectrometry setup in backscattered geometry was configured to measure the wavelength dependent reflectance from the biofilms at prescribed angles ranging from 37° to 48° (Supplemental Figure 1). The incident white light source was focused onto the biofilm and the backscattered light from the biofilm was collected with an optical fiber set to 8 degrees from the excitation. The biofilm was mounted vertically on a rotation stage to provide variable excitation angle with respect to the fixed optical setup. The collected signal was analyzed using an Ocean Optics spectrometer. Ambient room light was automatically subtracted from the sample acquisitions. Collected spectra showed sharp angle-dependent reflectance bands, suggesting constructive and coherent reflection through the biofilm.

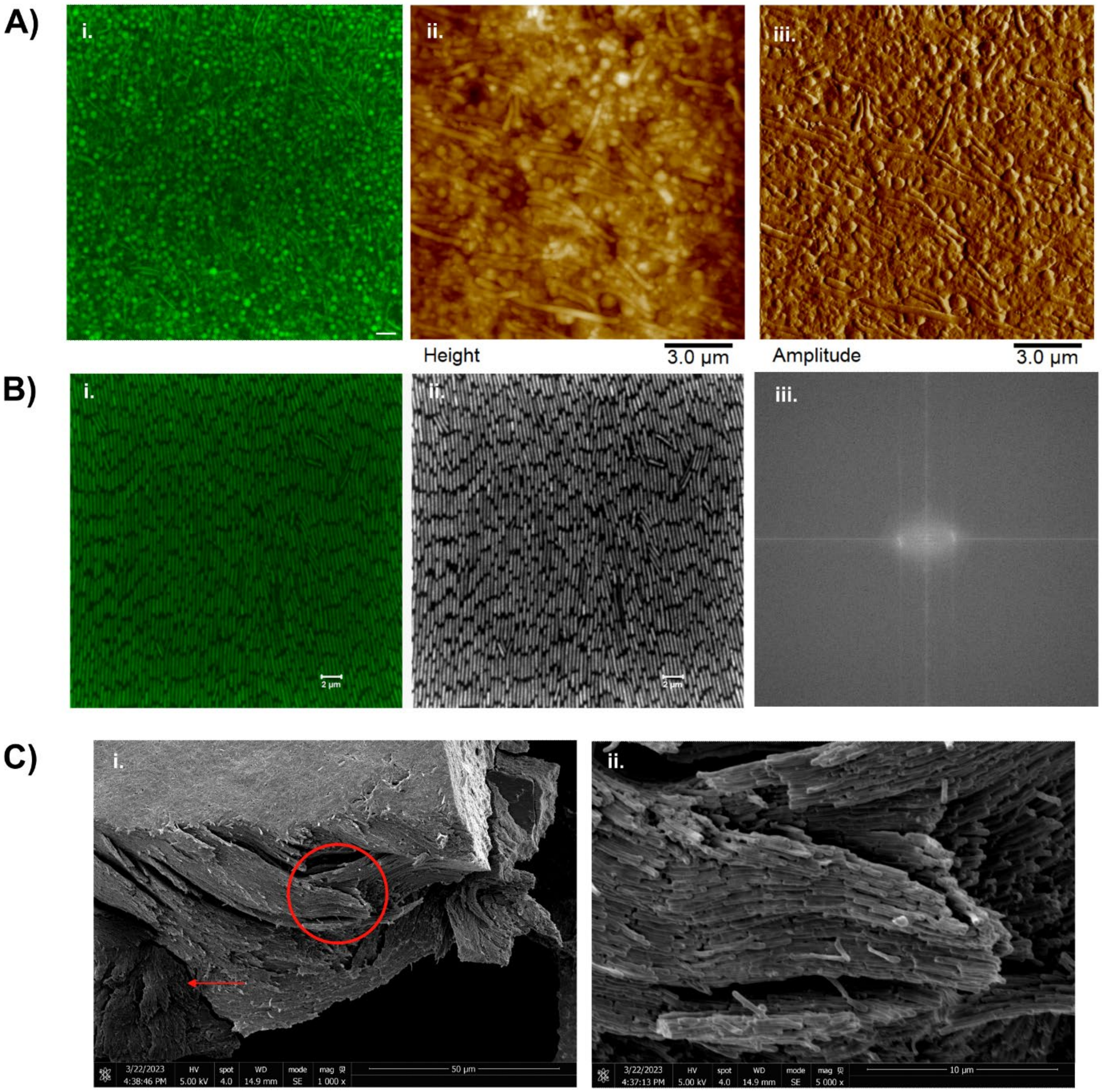

Supplemental Figure 2. (A) Images of *C. lytica* biofilm near the supporting agar showing an increased number of spherical cells. SYTO9 stained biofilm confocal fluorescence microscopy image (i.) Height (ii.) and amplitude (iii.) atomic force microscopy (AFM) images. **B)** Confocal microscopy image of upper region of SYTO9 stained iridescent biofilm (i.) with its 8-bit greyscale image (ii.) and FFT (iii.) showing the periodic arrangement of *C. lytica*. **(C)** Lateral images of biofilms, acquired using scanning electron microscopy (SEM) show that ordering occurs through the full thickness of the biofilms which ranges from 15 to 60 µm. Typical grain boundary (i, red arrow) where sections of the biofilm deviate in orientation though cells within each section are aligned. Next image (ii) is a magnification of the circled region in (i).

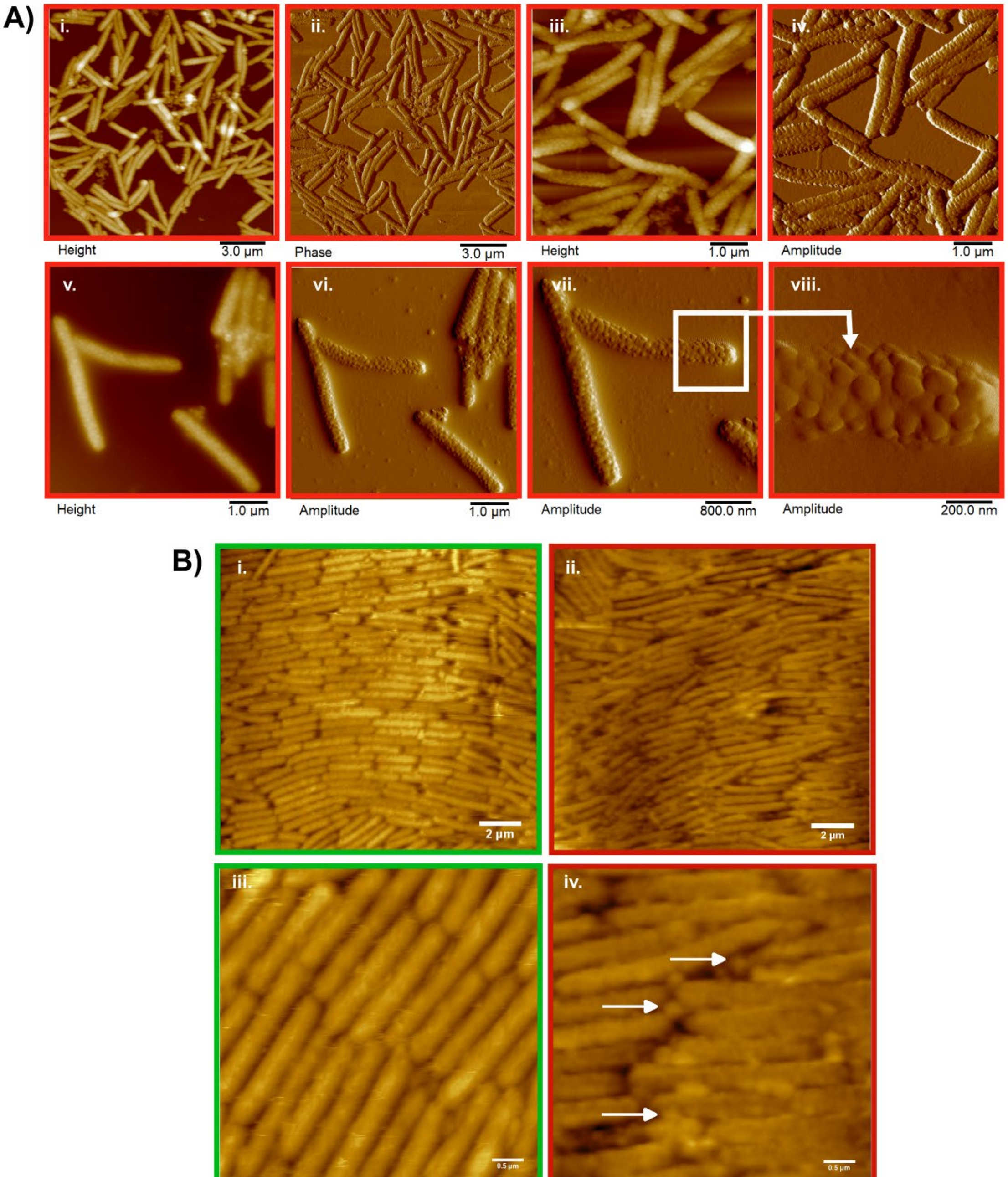

Supplemental Figure 3. Additional Imaging Data. (A) AFM images (height, phase or amplitude as indicated) of cells from red region at various magnifications, showing that red cells are covered by outer membrane vesicles. **(B)** Localized regions of fixed biofilms were also imaged in water by AFM for comparison. Consistent with the data presented previously, cells from green **(i, iii)** and red **(ii, iv)** iridescent regions are similarly planar, packed and arranged end-to-end. However, spherical protrusions, presumably vesicles, are mostly present in the images of the cells from the red region (arrows). Cells were prepared using the pick-up method and imaged in water.

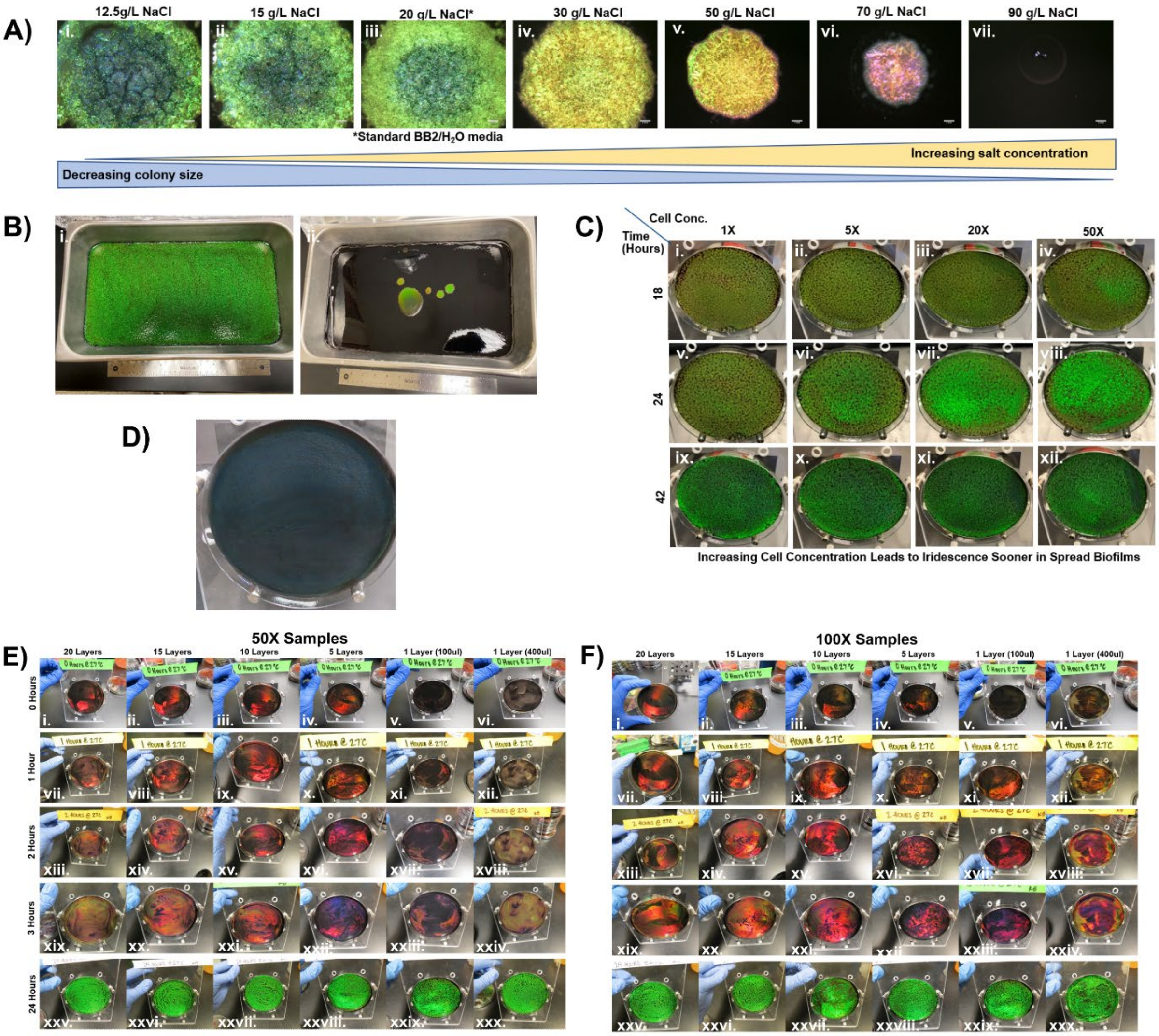

Supplemental Figure 4. (A) Increasing concentrations of NaCl in the media leads to a red shift in the color reflected by inoculated 4-day biofilms (i-vii). Colony dimensions are inversely proportional to the salinity, an indication that the cells may be experiencing stress. (scale bar = 1mm) **(B)** When the inoculum is increased proportionately and dispersed over the growth substrate, the biofilm can be scaled to cover a larger surface (i). As before, without dispersing the cells, the bacteria expand gradually from the site of inoculation and do not cover the surface as quickly (ii). **(C)** Concentrating the inoculum before dispersing further reduces the time to iridescence in ambient temperatures(i-xii) to within 24 hours. **(D)** 50X Concentrated inoculum dispersed on plates containing only MB, water, and 1% agar leads to blue biofilms after 2 days at 27°C. **(E, F)** 50X and 100X concentrated cells (applied serially) organize into iridescent biofilms within 24 hours though intermediate colors appear sooner in the latter.

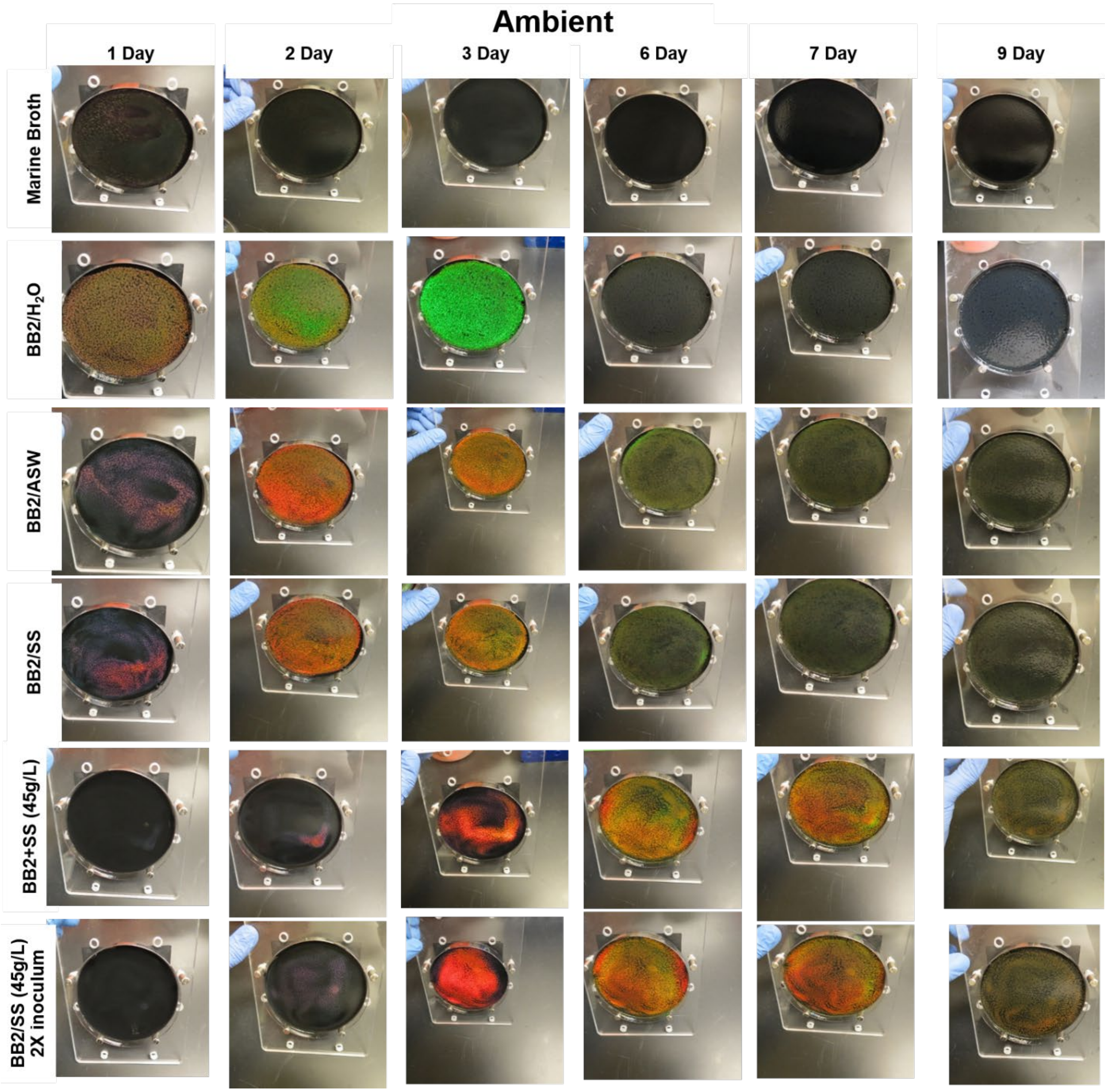

Supplemental Figure 5. Biofilm Reflections in Ambient Temperatures Modulate Over Time. Intervening colors can be reached by modulating temperature and the salinity of the growth media. Fast biofilms were made and allowed to develop in ambient conditions. The aggregated panel shows the color profile for various media over 8 days.

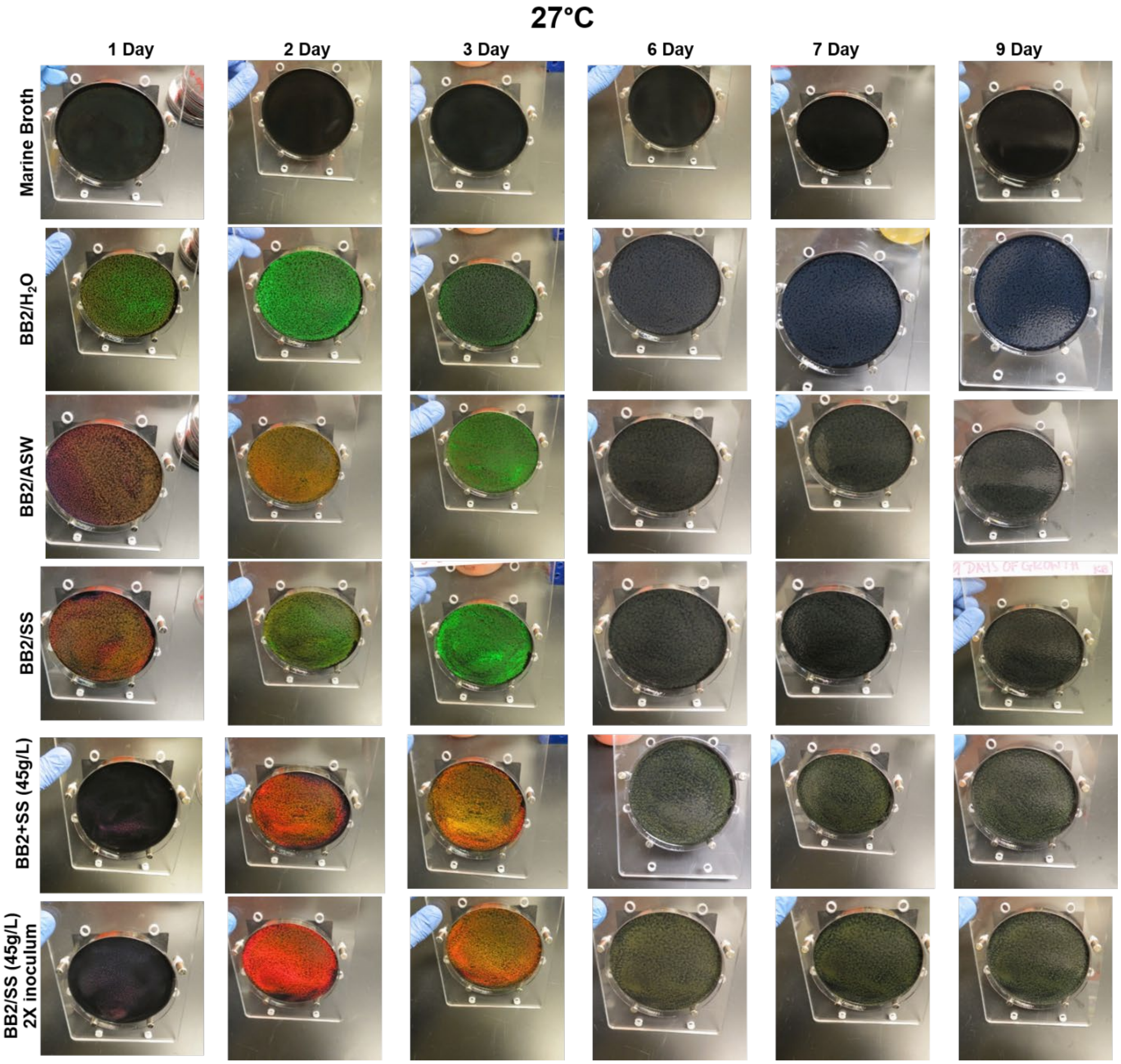

Supplemental Figure 6. Biofilm Reflections at 27°C Also Modulate Over Time. Increasing the temperature shifts color development temporally. The aggregated panel shows the color profile for various media over 8 days.

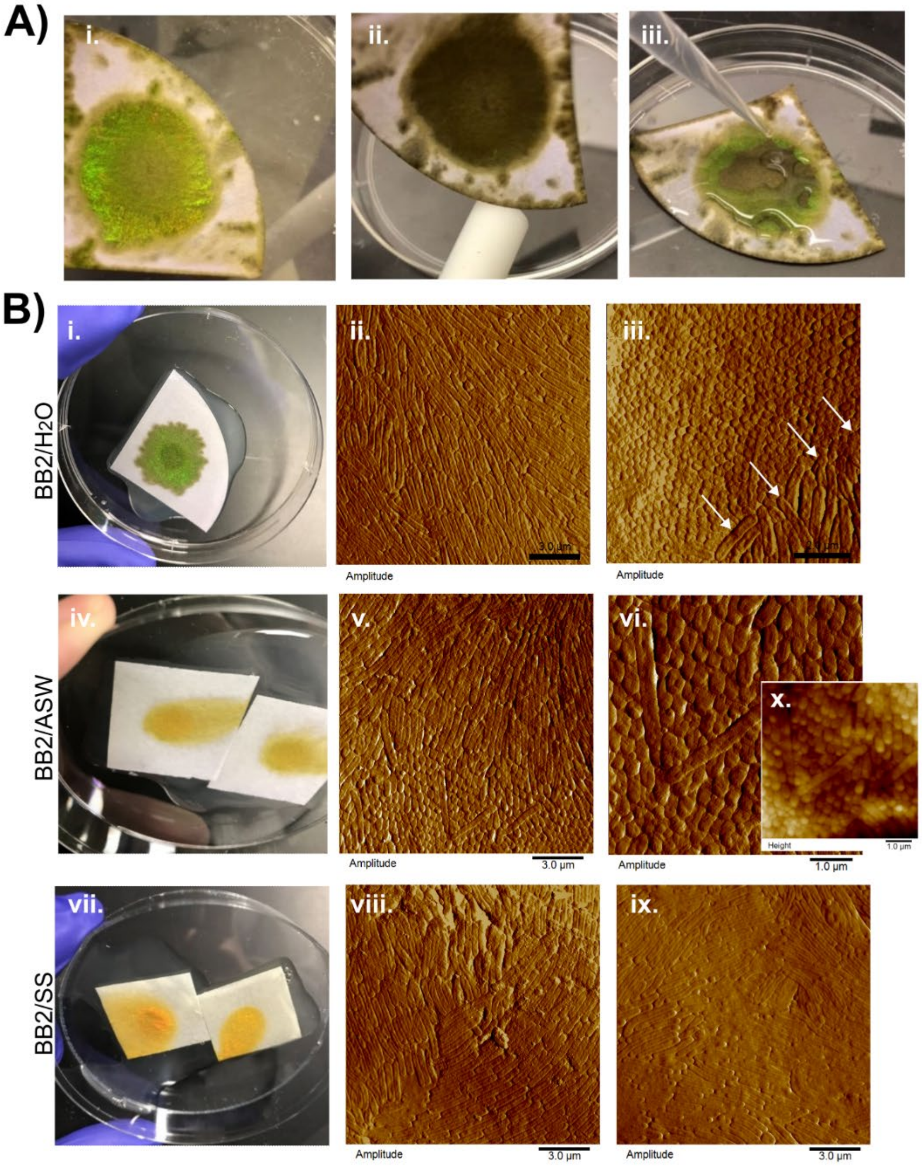

Supplemental Figure 7. (A) Videos showing that fixed paper associated biofilms (PABs) retain their iridescence after crosslinking with glutaraldehyde (i). However, they lose their iridescence after drying (ii). Rehydration restores the iridescence immediately (iii). The PABs facilitate handling during characterization and processing. **(B)** AFM of paper biofilms grown on BB2/H_2O,(i-iii), BB2/ASW (iv-vi) and BB2/SS (vii-ix). Grain boundaries are visible as are domains of cells having alternative orientations (iii, arrows). Inset (x) is a height image of the region shown in vi. Note the cell boundaries are poorly defined when biofilms are grown on BB2/SS (viii, ix). Cells are believed to be obscured by layers of vesicles.

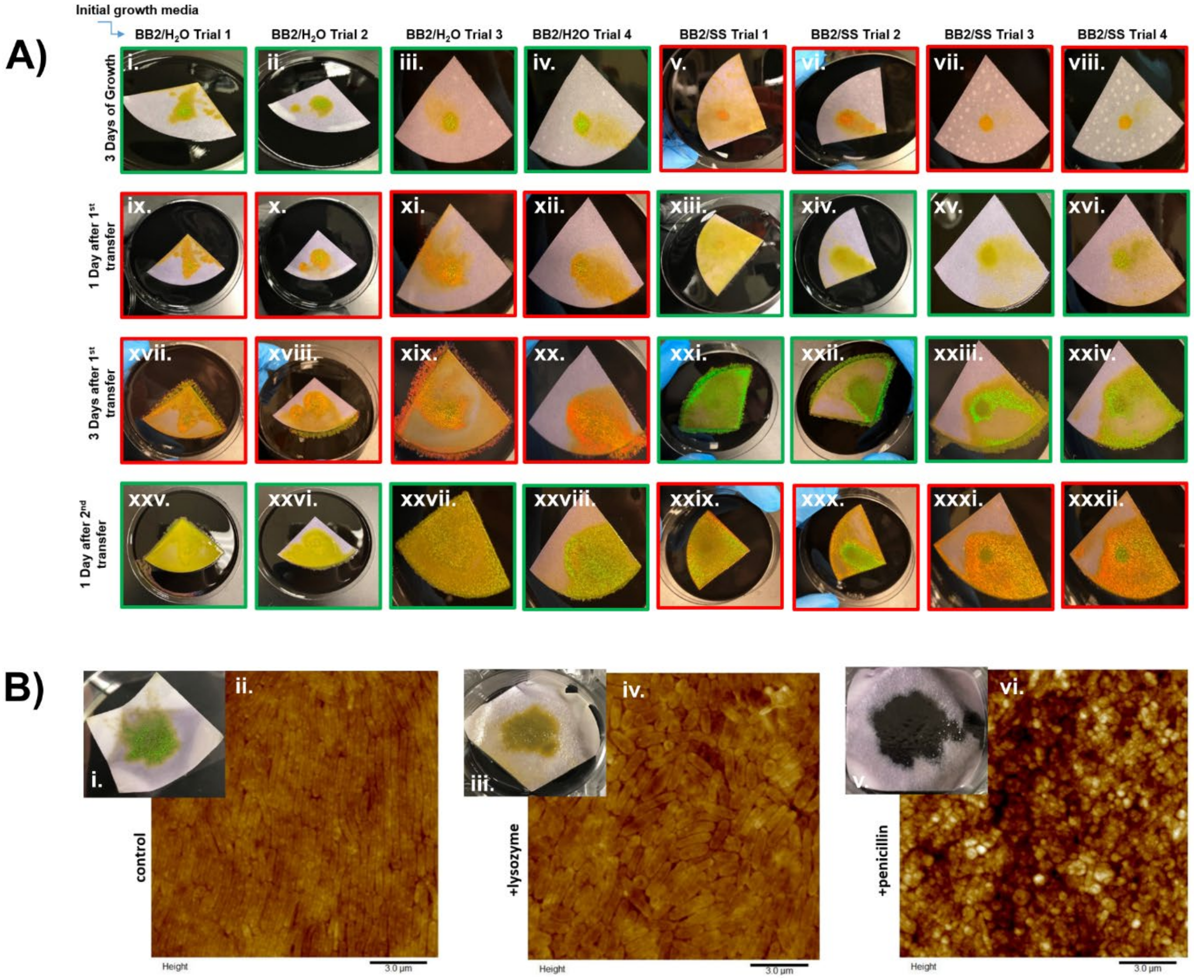

Supplemental Figure 8. (A-B) PABs grown in ambient conditions. **(A)** Living PABs retain their ability to respond to environmental cues. PABs from BB2/H_2O agar reflect mostly green (i-iv) until they are moved to BB2/SS plates (ix-xii, xvii-xx) where reflection is shifted red. Similarly, PABs originating on BB2/SS agar plates are red (v-viii) but change to green when placed on BB2/H_2O (xiii-xvi, xxi-xxiv). In both cases, biofilms are able to revert back to original color when returned to the original media condition (xxv-xxviii, xxix-xxxii), indications of their ability to sense and respond. **(B)** Additives such as salts, enzymes and small molecules in the agar plates can be used to alter biofilm properties in ambient growth conditions. Photographs of paper associated biofilms grown on BB2/H_2O agar plates augmented with buffer (i), 20 mg/ml lysozyme (iii), or 0.02 mg/ml penicillin (v). AFM images of the PABs in i-iii after fixation show that buffer control cells (ii) have typical rod-shaped morphologies whereas those treated with lysozyme (iv) and penicillin (vi) were enlarged and converted to spheres, respectively. Together these results confirm that corresponding changes in biofilm color at the macroscale are a consequence of changes in cell morphologies.

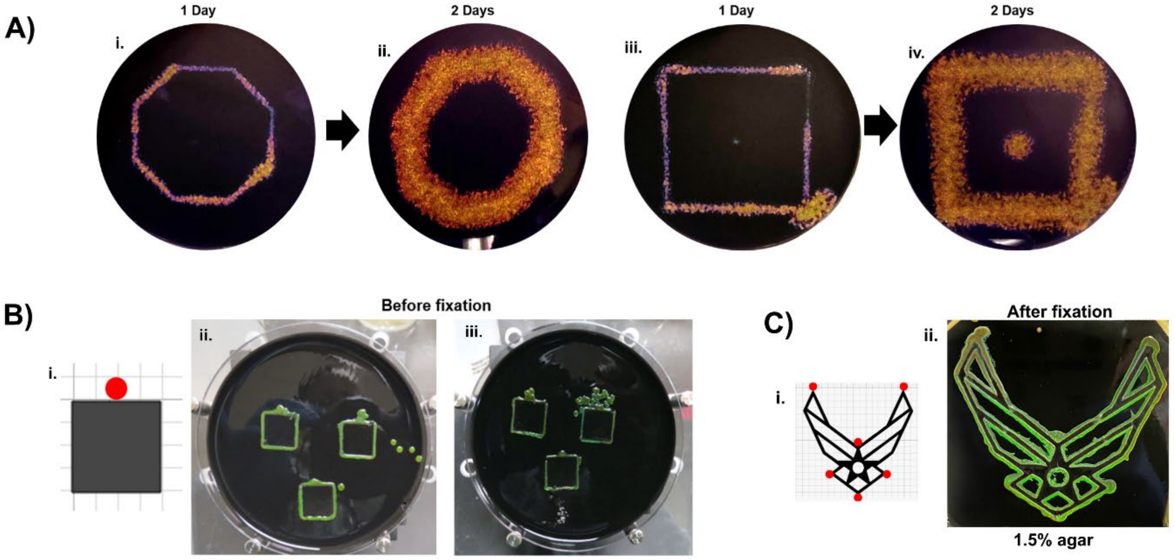

Supplemental Figure 9. Printing with *C. lytica* biofilms. (A) 3D printed designs containing *C. lytica* were generated on agar containing BB2/SS using an Allevi 3 Bioprinter setup (i-iv). As previously shown for dispersed biofilms, increased salinity red shifts the *C. lytica* ink's reflection. **(B)** Live *C. lytica* trace the edges of square templates (i) where the red circle indicates the site of inoculation. Cells behave like a self-printing bioink to write squares on agar (ii, iii). Increasing the agar concentration from 1.0% (ii) to 1.5% (iii) reduces the width of the traces. **(C)** Template of a complex pattern (i) and its BACTraced counterpart (ii) after fixation showing that the iridescent pattern can be preserved.

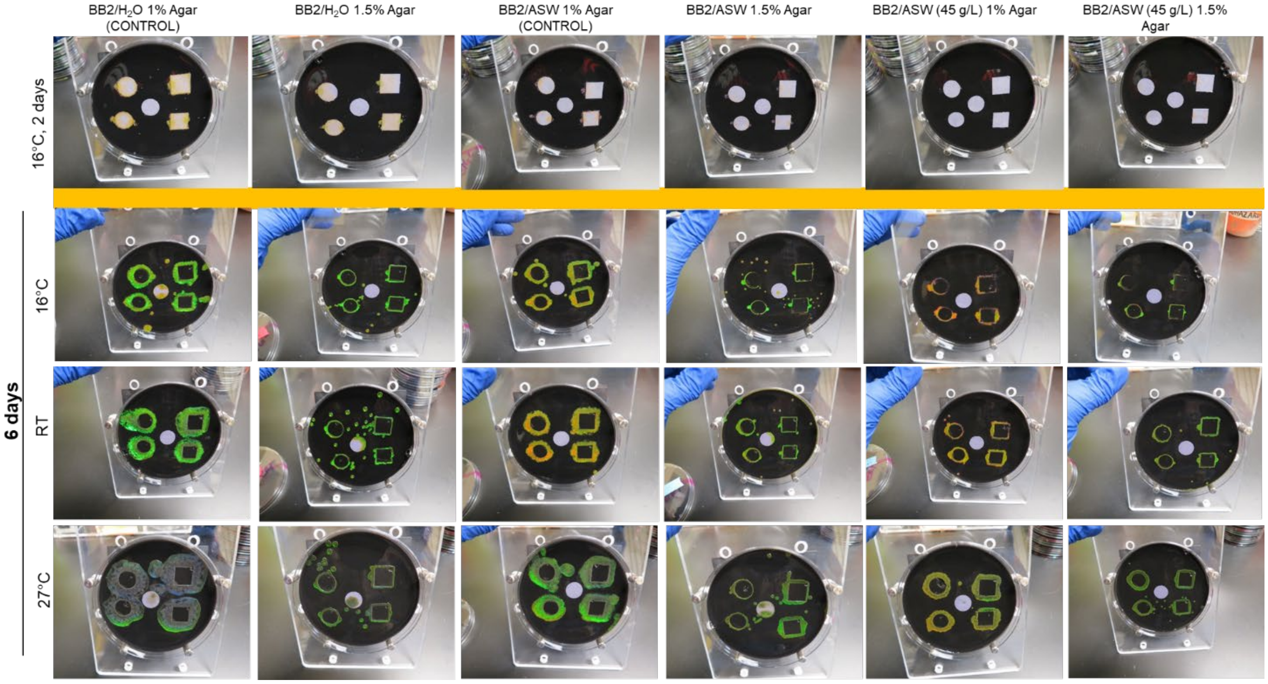

Supplemental Figure 10. Tuning BACTracing Using Growth Conditions. Both growth temperature and agar concentration can be used to influence BACTracing. Trace widths narrow significantly in conditions associated with slower growth or diminished gliding.

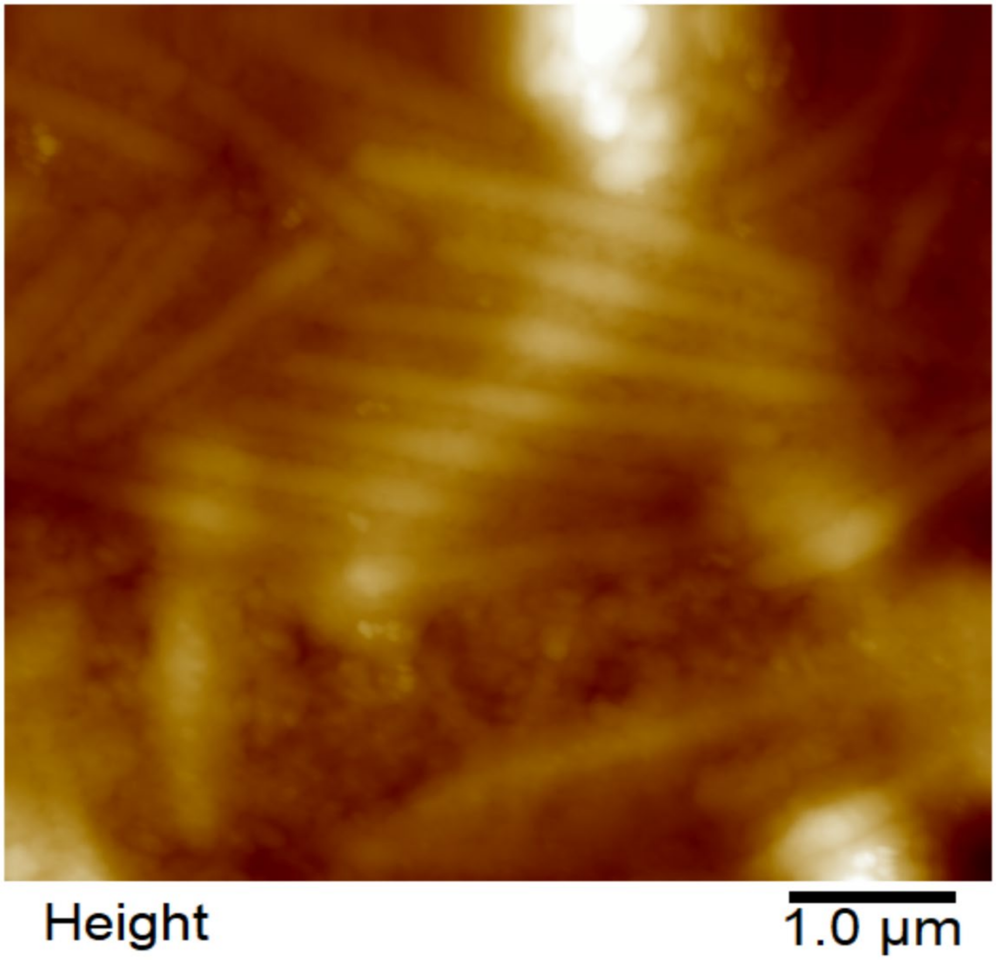

Supplemental Figure 11. AFM image of cells isolated from the red region of biofilm showing apparent intervening vesicles.

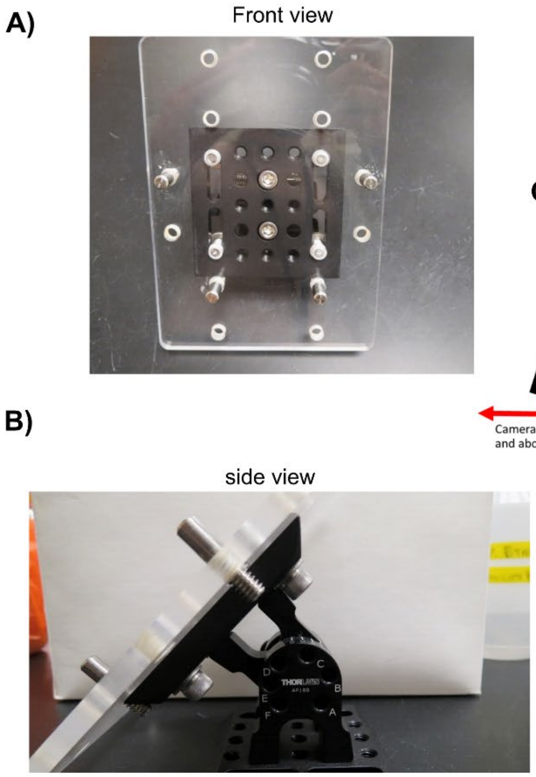

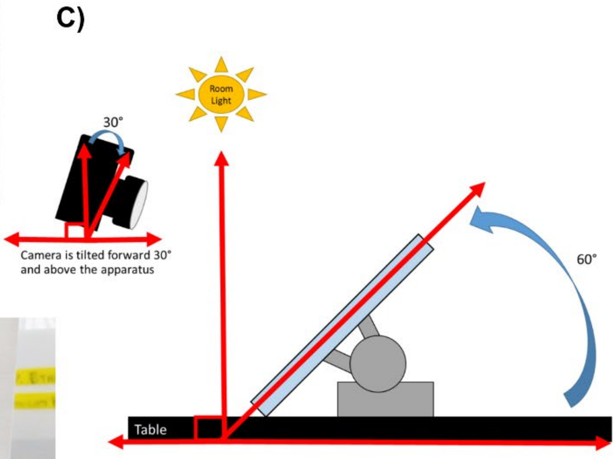

Supplemental Figure 12: Augmented adjustable angle mounting plate. (A) Front view. **(B)** Side view. **(C)** Schematic illustrating the setup used to capture photos of iridescent biofilms.

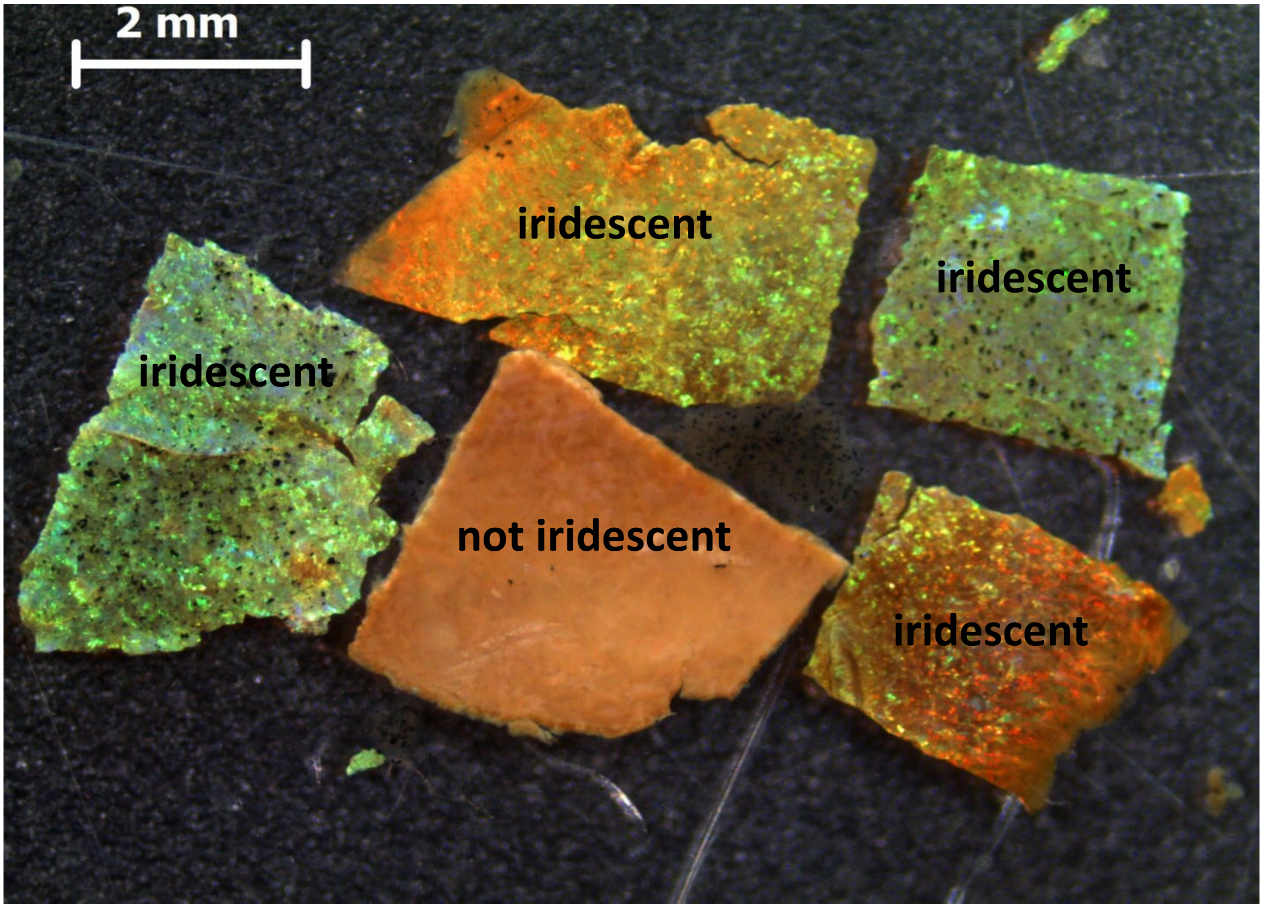

Supplemental Figures 13. Optical image of biofilms submerged in water. After removing fixed biofilms from the agar using a gentle agitation in a hot water, they maintain their integrity, are stable in water and retain their distinct iridescence.

Supplementary Figure Legends:

Supplemental Figure 1. Spectrometry Measurements. An *ad hoc* spectrometry setup in backscattered geometry was configured to measure the wavelength dependent reflectance from the biofilms at prescribed angles ranging from 37° to 48° (Supplemental Figure 1). The incident white light source was focused onto the biofilm and the backscattered light from the biofilm was collected with an optical fiber set to 8 degrees from the excitation. The biofilm was mounted vertically on a rotation stage to provide variable excitation angle with respect to the fixed optical setup. The collected signal was analyzed using an Ocean Optics spectrometer. Ambient room light was automatically subtracted from the sample acquisitions. Collected spectra showed sharp angle-dependent reflectance bands, suggesting constructive and coherent reflection through the biofilm.

Supplemental Figure 2. (A) Images of *C. lytica* biofilm near the supporting agar showing an increased number of spherical cells. SYTO9 stained biofilm confocal fluorescence microscopy image (i.) Height (ii.) and amplitude (iii.) atomic force microscopy (AFM) images. **B)** Confocal microscopy image of upper region of SYTO9 stained iridescent biofilm (i.) with its 8-bit greyscale image (ii.) and FFT (iii.) showing the periodic arrangement of *C. lytica*. **(C)** Lateral images of biofilms, acquired using scanning electron microscopy (SEM) show that ordering occurs through the full thickness of the biofilms which ranges from 15 to 60 µm. Typical grain boundary (i, red arrow) where sections of the biofilm deviate in orientation though cells within each section are aligned. Next image (ii) is a magnification of the circled region in (i).

Supplemental Figure 3. Additional Imaging Data. (A) AFM images (height, phase or amplitude as indicated) of cells from red region at various magnifications, showing that red cells are covered by outer membrane vesicles. **(B)** Localized regions of fixed biofilms were also imaged in water by AFM for comparison. Consistent with the data presented previously, cells from green **(i, iii)** and red **(ii, iv)** iridescent regions are similarly planar, packed and arranged end-to-end. However, spherical protrusions, presumably vesicles, are mostly present in the images of the cells from the red region (arrows). Cells were prepared using the pick-up method and imaged in water.

Supplemental Figure 4. (A) Increasing concentrations of NaCl in the media leads to a red shift in the color reflected by inoculated 4-day biofilms (i-vii). Colony dimensions are inversely proportional to the salinity, an indication that the cells may be experiencing stress. (scale bar = 1mm) **(B)** When the inoculum is increased proportionately and dispersed over the growth substrate, the biofilm can be scaled to cover a larger surface (i). As before, without dispersing the cells, the bacteria expand gradually from the site of inoculation and do not cover the surface as quickly (ii). **(C)** Concentrating the inoculum before dispersing further reduces the time to iridescence in ambient temperatures(i-xii) to within 24 hours. **(D)** 50X Concentrated inoculum dispersed on plates containing only MB, water, and 1% agar leads to blue biofilms after 2 days at 27°C. **(E,F)** 50X and 100X concentrated cells (applied serially) organize into iridescent biofilms within 24 hours though intermediate colors appear sooner in the latter.

Supplemental Figure 5. Biofilm Reflections in Ambient Temperatures Modulate Over Time. Intervening colors can be reached by modulating temperature and the salinity of the growth media. Fast biofilms were made and allowed to develop in ambient conditions. The aggregated panel shows the color profile for various media over 8 days.

Supplemental Figure 6. Biofilm Reflections at 27°C Also Modulate Over Time. Increasing the temperature shifts color development temporally. The aggregated panel shows the color profile for various media over 8 days.

Supplemental Figure 7. (A) Videos showing that fixed paper associated biofilms (PABs) retain their iridescence after crosslinking with glutaraldehyde (i). However, they lose their iridescence after drying (ii). Rehydration restores the iridescence immediately (iii). The PABs facilitate handling during characterization and processing. **(B)** AFM of paper biofilms grown on BB2/H_2O,(i-iii), BB2/ASW (iv-vi) and BB2/SS (vii-ix). Grain boundaries are visible as are domains of cells having alternative orientations (iii, arrows). Inset (x) is a height image of the region shown in vi. Note the cell boundaries are poorly defined when biofilms are grown on BB2/SS (viii, ix). Cells are believed to be obscured by layers of vesicles.

Supplemental Figure 8. (A-B) PABs grown in ambient conditions. **(A)** Living PABs retain their ability to respond to environmental cues. PABs from BB2/H_2O agar reflect mostly green (i-iv) until they are moved to BB2/SS plates (ix-xii, xvii-xx) where reflection is shifted red. Similarly, PABs originating on BB2/SS agar plates are red (v-viii) but change to green when placed on BB2/H_2O (xiii-xvi, xxi-xxiv). In both cases, biofilms are able to revert back to original color when returned to the original media condition (xxv-xxviii, xxix-xxxii), indications of their ability to sense and respond. **(B)** Additives such as salts, enzymes and small molecules in the agar plates can be used to alter biofilm properties in ambient growth conditions. Photographs of paper associated biofilms grown on BB2/H_2O agar plates augmented with buffer (i), 20 mg/ml lysozyme (iii), or 0.02 mg/ml penicillin (v). AFM images of the PABs in i-iii after fixation show that buffer control cells (ii) have typical rod-shaped morphologies whereas those treated with lysozyme (iv) and penicillin (vi) were enlarged and converted to spheres, respectively. Together these results confirm that corresponding changes in biofilm color at the macroscale are a consequence of changes in cell morphologies.

Supplemental Figure 9. Printing with *C. lytica* biofilms. (A) 3D printed designs containing *C. lytica* were generated on agar containing BB2/SS using an Allevi 3 Bioprinter setup (i-iv). As previously shown for dispersed biofilms, increased salinity red shifts the *C. lytica* ink's reflection. **(B)** Live *C. lytica* trace the edges of square templates (i) where the red circle indicates the site of inoculation. Cells behave like a self-printing bioink to write squares on agar (ii, iii). Increasing the agar concentration from 1.0% (ii) to 1.5% (iii) reduces the width of the traces. **(C)** Template of a complex pattern (i) and its BACTraced counterpart (ii) after fixation showing that the iridescent pattern can be preserved.

Supplemental Figure 10. Tuning BACTracing Using Growth Conditions. Both growth temperature and agar concentration can be used to influence BACTracing. Trace widths narrow significantly in conditions associated with slower growth or diminished gliding.

Supplemental Figure 11. AFM image of cells isolated from the red region of biofilm showing apparent intervening vesicles.

Supplemental Figure 12: Augmented adjustable angle mounting plate. (A) Front view. **(B)** Side view. **(C)** Schematic illustrating the setup used to capture photos of iridescent biofilms.

Supplemental Figures 13. Optical image of biofilms submerged in water. After removing fixed biofilms from the agar using a gentle agitation in a hot water, they maintain their integrity, are stable in water and retain their distinct iridescence.
